# The Diagnosis of Tuberculous Laryngitis

**Published:** 1907-08-31

**Authors:** 


### Laryngology and Rhinology.

### THE DIAGNOSIS OF TUBERCULOUS LARYNGITIS.

Now that it is generally realised that tuberculous laryngitis is often curable by local and general measures, provided always that it be treated in an early stage, the routine laryngoscopic examination of consumptives and the early diagnosis of laryngeal tuberculosis have assumed great practical importance.

In most cases, where the question of diagnosis arises, it is already known that the patient is suffering from phthisis, but it also happens, and not very uncommonly, that the suspicion of tuberculosis is first aroused by the laryngoscopic appearance, and in such a case the general signs, such as the temperature and the sputum, together with the physical examination of the lungs, are of great value as corroborative evidence. But it must be remembered that all laryngeal lesions in a consumptive patient are not tuberculous. On the other hand, tuberculous laryngitis may occur in an early stage of phthisis when the physical signs are very indefinite, and in cases of advanced laryngeal disease the pulmonary signs are often masked to an extraordinary extent.

The idea that anæmia of the larynx is distinctive of tuberculous disease requires qualification; tuberculous ulcers are generally pale, and so are the large swollen arytenoids, but the unaffected parts of the larynx have no characteristic colour, and many of the lesions, especially on the cords and epiglottis, are of a deep red tint; pallor of the larynx depends on general anæmia, which is common, but by no means universal, in phthisis. When there is hoarseness, the character of the voice is of some value for diagnosis; it is a weak voice with little expiratory effort and quite different from the raucous voice of syphilis or the husky voice of a new growth. The tuberculous lesion may consist of infiltration or ulceration, and the latter has certain distinctive characters; the edge is extremely ill-defined and has no surrounding zone of injection, the base is set with pale granulations which are usually small and give to the ulcer a "speckly" appearance, but in the interarytenoid region they are apt to form large, soft, irregular masses; the ulcer rarely extends much in depth except in the neighbourhood of the vocal process, where it often involves the cartilage and forms by necrosis a huge cavity.

Though the entire organ is involved at a late stage, the lesions may begin at various parts of the larynx; Three common types may be distinguished in which the cords, the interarytenoid region, and the upper aperture are respectively involved.

(1) Tuberculous infection of the vocal cord may at first appear as a simple redness; swelling may be slight or quite extreme; in the latter case the site of opposition of the other cord is sometimes marked by a groove, and this cleft is very characteristic. The cord may be covered with granulations, which appearance is almost pathognomonic, and, as mentioned above, an ulcer is common at the vocal process and frequently extends into the cartilage. Catarrhal inflammation and syphilis are the diseases which most often imitate this chordal form of tuberculous laryngitis. When the lesion has produced no more than simple redness of both cords it is indistinguishable from catarrh, it can only be said that a catarrh which persists in spite of simple treatment and vocal rest must be regarded with suspicion; but marked redness confined to one cord is never due to catarrh, though it may be caused by tubercle, syphilis, epithelioma, or traumatism; a patchy congestion is in favour of tubercle rather than of simple catarrh, and ulceration does not occur in the latter affection. Syphilis attacks the anterior part of the glottis more often than tuberculosis; syphilitic ulcers have defined red edges and a smooth non-granular base; there is a greater tendency to scarring and the lesions are more widely distributed and tend to attack the fauces, palate, and pharynx. Epithelioma rarely imitates tuberculous disease of the vocal cord, although the converse occurs in certain cases where tuberculosis attacks the vocal cord of elderly individuals; but the differential diagnosis of these important cases will be more usefully discussed on a subsequent occasion, when epithelioma is under consideration.

(2) In the interarytenoid region tuberculous infiltration is often found; in the earliest stage it takes the form of a soft swelling of a peculiar velvety aspect; papillary excrescences are common and may reach a large size, ulceration often occurs, and is of diagnostic value, as other forms of ulcer are rare in this situation. This type has to be distinguished from the interarytenoid thickening which occurs in the pachydermatous form of chronic laryngitis; the latter is firm, regular, and symmetrical, and usually shows a central depression, whereas tuberculous interarytenoid infiltration is typically soft, translucent, and irregular.

(3) The form of tuberculosis affecting the upper aperture is in general easy of diagnosis; the arytenoids are usually attacked first, and the resulting smooth translucent pyriform swellings are almost pathognomonic. This swelling is nearly always bilateral, in œdematous laryngitis symmetrical swelling of the upper aperture occurs, but the affection is acute and differs from tuberculous laryngitis in its entire aspect. The œdema of arytenoid perichondritis is unilateral with complete fixation of the cord; a gumma of the arytenoid is likewise unilateral, firm and solid, and usually breaks down to form the typical deep "crateriform" ulcer; a malignant growth in this region is firm and hard, red and angry, it ulcerates early and resembles syphilis rather than tuberculosis, for tuberculous ulceration here occurs late and is seldom extensive. The epiglottis is rarely attacked by tuberculosis until the rest of the larynx is extensively affected; the infiltration is smooth and round, and ulceration occurs on the laryngeal aspect; the "turban-shaped" appearance of the tuberculous upper aperture is quite distinctive. Syphilis, on the contrary, often reaches the larynx from the fauces, attacks the epiglottis early, and produces marked ulceration which begins on the lingual aspect and frequently destroys the entire part.

